# E-cigarette awareness and use, among adult residents in Shanghai, China

**DOI:** 10.18332/tid/169559

**Published:** 2023-08-14

**Authors:** Jian Wang, Chen-Chen Xie, Xiao-Xian Jia, Kun Xu, Zheng-Yang Gong, Yuan-Qiao Sun, Jing-Rong Gao, Yuan Ding, Zhi-Yong Huang, De Chen

**Affiliations:** 1 Shanghai Municipal Center for Health Promotion, Shanghai, China; 2 Shanghai Association of Tobacco Control, Shanghai, China; 3 Shanghai Development Promotion Centre of Undertakings for the Aged, Shanghai, China; 4 Shanghai Municipal Health Commission, Shanghai, China

**Keywords:** e-cigarettes, perception, adult, China, smoke

## Abstract

**INTRODUCTION:**

The widespread popularity of e-cigarettes is considered an important public health concern. However, only some studies have investigated the prevalence of e-cigarette use in Shanghai, China. Research on the perceived harmfulness of e-cigarettes and public support for e-cigarette regulations in China is limited. This study aimed to estimate e-cigarette awareness, prevalence, and associated factors among adults in Shanghai, China.

**METHODS:**

This study used data from a representative survey conducted in Shanghai, China, in 2019. The survey was conducted at 64 surveillance points in Shanghai, China, using a multistage, stratified, cluster-randomized sampling design, recruiting community-based Chinese adults aged ≥15 years. Based on the principles outlined in the Global Adult Tobacco Survey (GATS) China Project, data were collected by conducting face-to-face interviews in households. Of the 3200 selected households, 3060 people completed the individual survey. The overall response rate was 97.4%.

**RESULTS:**

In all, 72.3% of the respondents had heard of e-cigarettes. The respondents who had used e-cigarettes at some point in their life, used them in the last 12 months, and used them currently were 5.8%, 2.6%, and 1.3%, respectively. Among adult residents who had heard of e-cigarettes, 38.2% thought they were less harmful than traditional cigarettes. The respondents who perceived e-cigarettes as more harmful than traditional cigarettes were less likely to have ever used e-cigarettes (AOR=0.2; 95% CI: 0.1–0.5, p=0.0015) and more likely to support incorporating e-cigarettes into the regulation of smoking control (AOR=3.9; 95% CI: 1.8–8.6, p=0.0008).

**CONCLUSIONS:**

Our findings reveal that the awareness about e-cigarettes was high, and the prevalence of e-cigarette use was similar to the findings from previous studies in China. The harmful perception of e-cigarettes warrants further attention from public health practitioners.

## INTRODUCTION

Electronic cigarettes, also known as ‘e-cigarettes’, are the most common Electronic Nicotine Delivery Systems (ENDS). They are battery-powered devices that deliver smoke-like aerosol (i.e. vapor) that the user inhales^1,2^. E-cigarette sales worldwide are expected to grow to US$28.2 billion by 2027^3^.

Although the long-term health effects of e-cigarettes remain unclear, sufficient evidence suggests that they are unsafe^4-6^. Chronic exposure to e-cigarette aerosol during pregnancy has potentially harmful developmental effects on offspring^7,8^. Growing evidence from clinical and epidemiological studies indicates that e-cigarettes and their aerosol constituents can adversely affect the cardiovascular, brain development, and respiratory systems^9-13^.

E-cigarettes have become popular as they are perceived to be safer compared to traditional cigarette smoking. This public perception of safety have gained popularity among non-smokers^9^.

In China, national survey data showed that e-cigarette use among adults remains low but has increased substantially from 2015 to 2018^14^. Zhao et al.^15^ identified a significant increase in e-cigarette use among urban populations between 2015 and 2019. Two recent studies used city-level representative data to assess the awareness and use of e-cigarettes among urban residents in China^16,17^. However, these studies have yet to report data on e-cigarette use among adults in Shanghai, China. Shanghai is a municipality under the direct administration of the Chinese government. According to the 2020 population census in China, the population of Shanghai was 24.87 million^18^. The studies conducted in Shanghai may contribute to a better understanding of e-cigarette awareness and use among adult Chinese residents.

Various e-cigarette regulations have been implemented worldwide to reduce the potential risks of e-cigarettes. A total of 32 countries have banned ENDS^1^. If e-cigarettes are not banned, they must be regulated. The Regulation of the Shanghai Municipality on smoking control in public places was officially implemented on 1 March 2010 and was the first domestic tobacco control regulation promulgated by the Provincial People’s Congress. The first amendment to the regulations was implemented on 1 March 2017. Additionally, smoking is prohibited in indoor public places, workplaces, and public transport facilities in Shanghai. On 28 October 2022 inclusion of e-cigarettes within the scope of smoking prohibition in public places in Shanghai was approved and implemented. However, knowledge regarding the characteristics of e-cigarette prevalence and trends in e-cigarette use in Shanghai before e-cigarettes were incorporated into regulations remains limited.

Based on the knowledge and understanding outlined above, this study aimed to estimate e-cigarette awareness, prevalence, and associated factors among adults in Shanghai. Additionally, we examined the perceived harm and public support for incorporating e-cigarettes into the Regulation of Shanghai Municipality on smoking control to provide important baseline data before the implementing e-cigarette regulation in Shanghai.

## METHODS

### Study population and settings

We conducted a cross-sectional household survey in Shanghai, China, from June 2019 to September 2019. Eligible subjects were Chinese residents aged ≥15 years who resided in the selected household in the month prior to the survey date, excluding those collectively living in student dormitories, nursing homes, military camps, prisons, or hospitals^14,16-17^. A stratified four-stage cluster sampling design was implemented to produce representative citywide data based on the principles outlined in the GATS Sample Design Manual^19,20^.

In the first stage, 64 streets or towns were selected using the probability proportionate to size (PPS) sampling method. The measure of size (MOS) was the number of households. In the second stage, one urban neighborhood community (Ju-Wei-Hui in Mandarin) or rural village was selected from each of the selected streets or towns using the PPS method. The selected secondary sampling unit was partitioned into segments of around 750–1500 households, if the selected unit was of above 1500 households. One segment was randomly selected thereafter. In the third stage, 50 households from each neighborhood community or selected segment, were randomly selected from the household sampling frame created by mapping and listing. The total number of designated households was 3200. In the last stage, one individual was randomly selected from each of the 3200 participating households using simple random sampling.

The questionnaire was based on the Global Adults Tobacco Survey China Project^14^, with additional questions on the perception of e-cigarette harmfulness and support to incorporate e-cigarettes into the regulation of Shanghai Municipality on smoking control.

### Measures

*Sociodemographic characteristics*

Assessed sociodemographic characteristics included: gender, age group (15–24, 25–44, 45–64, ≥65 years), educational level (primary school or lower, junior high school, senior high school, college degree or higher), occupation (government employee, teacher, healthcare provider; factory, business, agriculture, and service industry employee; and not in the labor force, which included the unemployed, students, homemakers, and retired)^16^, annual household income in RMB (<30000, 30000–49999, 50000–99999, ≥100000) and smoking status (never tobacco smoker, current tobacco smoker, former tobacco smoker).

*Measures about e-cigarettes*

Awareness of e-cigarettes was measured by the question: ‘Have you ever heard of e-cigarettes?’. If the answer was ‘yes’, then they were asked ‘Where did you hear about electronic cigarettes?’^14^. Among those who had ever heard of e-cigarettes, the perception of harm around e-cigarettes was measured by the question: ‘Compared with traditional cigarettes, do you think e-cigarettes are less harmful, just as harmful, or more harmful?’. The question: ‘Do you agree with the incorporation of e-cigarettes into the Regulation of Shanghai Municipality on smoking control?’ was asked to learn about the support for e-cigarette regulation in smoke-free venues.

Questions such as ‘Have you ever used an electronic cigarette?’, ‘Have you ever used an electronic cigarette during the past 12 months?’ and ‘Do you use e-cigarettes currently?’ were asked to measure ever use, past 12 months use, and current use of e-cigarettes^14^.

### Statistical analysis

SAS V.9.4 was used for data processing and analysis. Owing to the complex sample design of the survey, each responding unit was assigned a unique survey weight that was used to produce estimates of the population parameters^19^. Population data from the Shanghai Municipal Bureau of Statistics were used for the post-stratification calibration adjustment. Descriptive statistics of percentages, including point estimates and 95% confidence intervals (CI), were calculated overall and by sociodemographic characteristics. Rao-Scott chi-squared tests and univariate logistic regression were conducted to compare the outcome variables by sociodemographic characteristics. Multivariable logistic regression was conducted to explore factors associated with awareness and ever use of e-cigarettes. Adjusted odds ratios (AORs) controlled for sociodemographic characteristics, including age, gender, education level, occupation, income, and smoking status, in the analysis of factors associated with awareness of e-cigarettes among all samples. Two models were built to assess factors related to the ever use of e-cigarettes. One model was the same as the AOR analysis of awareness of e-cigarettes. Another model was adjusted for the perceived harmfulness of e-cigarettes compared to traditional cigarettes among respondents who had heard of e-cigarettes, in addition to the factors adjusted in the preceding model. A two-tailed p<0.05 was considered statistically significant.

## RESULTS

### Demographic characteristics

Of the 3200 selected households, 59 empty households were eliminated, 3093 households completed the survey, and 3060 people completed the individual survey. The overall response rate was 97.4%. The weighted percentage estimate of people with senior high school education accounted for 26.5% and those with a college degree or higher were 37.7%. The current smoking prevalence was 19.7% (Table 1).

**Table 1:** Demographic characteristics among adult residents in Shanghai, China, 2019 (N=3060)

*Demographic characteristics*	*Unweighted number n*	*%*	*95 % CI*	*Weighted number n*
**Overall**	3060	100		21979700
**Gender**				
Male	1346	51.6	48.8–54.3	11332500
Female	1714	48.4	45.7–51.2	10647200
**Age** (years )				
15–24	106	12.8	10.1–15.5	2816300
25–44	849	41.4	38.1–44.7	9095700
45–64	1094	32.3	29.5–35.0	7091100
≥65	1011	13.5	11.2–15.9	2976600
**Education level**				
Primary school or lower	559	10.3	8.2–12.4	2261512
Junior high school	902	25.5	22.4–28.6	5582055
Senior high school	741	26.5	23.6–29.4	5805034
College degree or higher	848	37.7	33.3–42.1	8259009
**Occupation**				
Government employee, teacher, healthcare provider	238	11.0	8.1–13.9	2409634
Factory, business, service industry employee	1102	45.2	39.9–50.6	9899667
Not in the labor force	1707	43.7	38.7–48.8	9571064
**Income** (RMB )				
<30000	456	8.4	5.9–10.8	1841797
30000–49999	575	12.0	9.6–14.5	2639449
50000–99999	990	30.9	26.6–35.1	6787537
≥100000	751	35.1	30.3–39.9	7714346
Unwilling to disclose	288	13.6	9.0–18.2	2996571
**Smoking status**				
Never smoker	2259	72.8	70.1–75.5	15996813
Current smoker	530	19.7	17.4–21.9	4326771
Former smoker	271	7.5	6.2–8.8	1656116

RMB: 1000 Chinese Renminbi about US$140.

### Awareness of e-cigarettes among adult residents in Shanghai

A total of 72.3% of adults aged ≥15 years had heard of e-cigarettes (Table 2). Awareness of e-cigarettes was higher among adults aged <65 years, males, participants with education level higher than primary school, and those with an annual household income above RMB 50000. Current smokers were also more aware of e-cigarettes.

**Table 2:** Awareness, ever use, past 12 months use, and current use of e-cigarettes among adult residents in Shanghai, China, 2019 (N=3060)

*Variable*	*Awareness of e-cigarettes*		*Ever use of e-cigarettes*		*Past 12 months use of e-cigarettes*		*Current use of e-cigarettes*	
	*% (9 5 % CI )*							
**Overall**	72.3	68.7–75.8	5.8	4.5–7.1	2.6	1.7–3.6	1.3	0.6–1.9
**Gender**								
Male	78.5	74.8–82.3	10.5	8.0–13.0	4.6	2.8–6.4	2.2	1.0–3.4
Female	65.6	61.3–70.0	0.8	0.3–1.4	0.6	0.0–1.1	0.3	0.0–0.8
**Age (**years)								
15–24	87.9	81.2–94.6	8.5	1.8–15.2	5.3	0.6–10.0	4.1	0.0–8.2
25–44	84.0	79.4–88.6	7.8	5.6–10.0	4.0	2.3–5.7	1.3	0.4–2.2
45–64	62.2	57.4–67.0	3.9	2.7–5.1	0.9	0.2–1.6	0.6	0.0–1.2
≥65	45.8	39.6–52.1	1.7	0.9–2.6	0.3	0.0–0.6	0.3	0.0–0.6
**Education level**								
Primary school or lower	25.4	20.4–30.3	2.6	0.4–4.9	0.0	-	0.0	-
Junior high school	63.5	58.6–68.3	5.7	2.1–9.4	1.9	0.1–3.8	0.7	0.0–1.8
Senior high school	79.4	75.2–83.7	6.3	4.0–8.6	2.7	0.7–4.7	1.6	0.1–3.1
College degree or higher	86.2	81.9–90.5	6.4	4.1–8.7	3.8	2.0–5.5	1.8	0.5–3.1
**Occupation**								
Government employee, teacher, healthcare provider	82.2	74.9–89.6	4.1	0.9–7.4	2.9	0.0–6.0	1.3	0.1–2.5
Factory, business, service industry employee	76.5	71.7–81.3	8.1	5.5–10.6	3.4	1.7–5.0	1.6	0.4–2.8
Not in the labor force	65.6	61.0–70.2	4.0	2.6–5.4	1.8	0.4–3.2	1.0	0.1–1.9
**Income** (RMB)								
<30000	50.2	40.8–59.6	4.0	0.3–7.7	0.5	0.0–1.4	0.5	0.0–1.4
30000–49999	58.1	49.8–66.3	5.0	2.6–7.4	1.0	0.0–2.2	0.4	0.0–0.9
50000–99999	73.4	69.3–77.4	4.3	2.5–6.0	2.1	0.8–3.4	0.9	0.1–1.7
≥100000	80.5	76.5–84.6	7.2	4.6–9.9	3.5	1.8–5.3	1.5	0.5–2.5
Unwilling to disclose	74.5	68.8–80.2	7.6	2.9–12.3	4.3	0.0–8.8	3.0	0.0–6.8
**Smoking status**								
Never smoker	69.7	65.7–73.7	0.7	0.1–1.3	0.6	0.0–1.2	0.2	0.0–0.5
Current smoker	82.9	78.4–87.5	23.7	18.5–28.9	10.8	6.7–15.0	5.6	2.6–8.7
Former smoker	69.3	63.1–75.5	8.5	4.5–12.6	1.1	0.0–2.2	0.4	0.0–1.2

RMB: 1000 Chinese Renminbi about US$140.

After controlling for demographic characteristics and smoking status in Model A, the age groups of 15–24 and 25–44 years were associated with a higher rate of awareness of e-cigarettes. Residents with higher educational level were more likely to be aware of e-cigarette use. Respondents with household incomes ≥50000 had greater odds of being aware of e-cigarettes than those with incomes <30000. Current and former smokers were more likely to be aware of e-cigarettes than non-smokers. There was no association between e-cigarette awareness and gender or occupation (Table 3).

**Table 3:** Factors associated with awareness and ever use of e-cigarettes among adult residents of Shanghai, China, 2019

*Variable*	*Awareness of e-cigarettes (N=3060)*		*Ever use of e-cigarettes (N=3060)*		*Ever use of e-cigarettes (N=1896)*	
	*AOR^a^*	*95% CI*	*AORa*	*95% CI*	*AOR^b^*	*95 % CI*
**Gender**						
Male	1.1	0.8–1.6	1.1	0.3–3.7	1.0	0.3–3.6
Female (Ref.)						
**Age** (years)						
15–24	4.3	2.2–8.6	12.9	3.8–43.4	4.4	1.2–16.0
25–44	2.6	1.6–4.4	5.9	2.5–13.9	3.8	1.6–9.1
45–64	1.3	1.0–1.6	1.6	0.7–3.5	1.3	0.6–2.8
≥65 (Ref. )						
**Education level**						
Primary school or lower (Ref.)						
Junior high school	3.9	2.9–5.3	1.4	0.5–4.3	0.8	0.3–2.8
Senior high school	7.4	5.3–10.3	1.0	0.4–3.0	0.7	0.2–2.3
College degree or higher	9.5	6.3–14.5	1.2	0.4–3.8	0.7	0.2–2.4
**Occupation**						
Government employee, teacher, healthcare provider	0.8	0.4–1.5	0.6	0.2–1.7	0.6	0.2–1.7
Factory, business, service industry employee	0.9	0.6–1.2	1.0	0.5–1.9	0.9	0.5–1.8
Not in the labor force (Ref.)						
**Income** (RMB)						
<30000 (Ref.)						
30000–49999	1.3	0.8–1.9	1.2	0.4–3.9	1.2	0.3–4.1
50000–99999	1.8	1.2–2.6	1.0	0.3–3.0	0.9	0.3–2.9
≥100000	1.8	1.2–2.7	1.6	0.6–4.9	1.3	0.5–3.7
Unwilling to disclose	1.5	1.0–2.2	1.9	0.6–6.2	1.9	0.5–7.0
**Smoking status**						
Never smoker (Ref.)						
Current smoker	2.7	1.7–4.2	62.9	19.2–206.0	63.5	18.5–217.4
Former smoker	1.8	1.2–2.7	29.7	7.9–112.0	26.0	7.0–95.9
**Perceived harmfulness compared with traditional cigarettes**						
E-cigarettes less harmful (Ref.)						
E-cigarettes similarly harmful					0.5	0.3–1.0
E-cigarettes more harmful					0.2	0.1–0.5
Do not know					0.3	0.1–0.5

AOR: adjusted odds ratio.

a All samples, adjusted for gender, age, education, occupation, income, and smoking status.

b Among respondents who had heard of e-cigarettes, adjusted for gender, age, education level, occupation, income, smoking status, and perceived harmfulness. RMB: 1000 Chinese Renminbi about US$140.

Regarding the information sources about e-cigarettes, the most common source was friends (56.3%), followed by the internet (44.9%) and television (40.7%). The residents who were younger, employed, and had higher level of education were more likely to learn about e-cigarettes on the internet (p<0.0001).

### Perception of e-cigarette harmfulness and support to incorporate e-cigarettes into the regulation of Shanghai Municipality on smoking control

**Figure 1:**
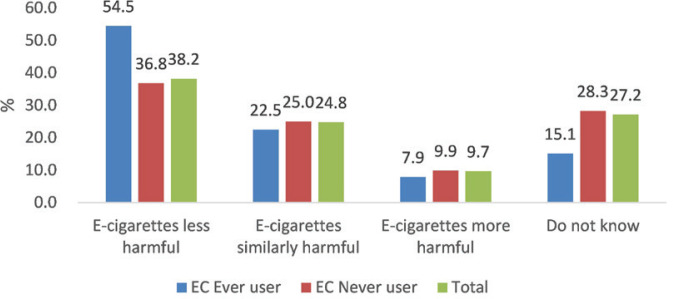
Perceived harm of e-cigarettes relative to traditional cigarettes by ever use of e-cigarettes in Shanghai, China, 2019 (N=1896)

Among the adult residents who had heard of e-cigarettes, 38.2% thought that e-cigarettes were less harmful than traditional cigarettes, 24.8% thought they were similarly harmful, and 27.2% were unaware of their harmfulness (Table 4 and Figure 1). More e-cigarette users (54.5%) than never e-cigarette users (36.8%) perceived e-cigarettes as less harmful than traditional cigarettes (p=0.006). The perception of e-cigarette harmfulness differed among age groups (p<0.0001). The rate of perceiving e-cigarettes as less harmful than traditional cigarettes ranged from 55.6% among participants aged 15–24 years to 27.6% among participants aged ≥65 years.

**Table 4:** Perception of e-cigarettes and support for e-cigarette use regulation by ever use of e-cigarettes among respondents who had heard of e-cigarettes in Shanghai, China, 2019 (N=1896)

*Items*	*Ever use of e-cigarettes (N=117)*		*Never use of e-cigarettes (N=1779)*		*Total*		*p*
	*% (95% CI)*						
**Perceived harmfulness compared with traditional cigarettes**							
E-cigarettes less harmful	54.5	44.2–64.8	36.8	32.8–40.8	38.2	34.4–42.0	0.006
E-cigarettes similarly harmful	22.5	13.8–31.3	25.0	21.9–28.1	24.8	21.9–27.8	
E-cigarettes more harmful	7.9	2.9–12.8	9.9	7.8–12.0	9.7	7.9–11.6	
Do not know	15.1	7.3–22.9	28.3	24.9–31.6	27.2	24.1–30.3	
**Support to incorporate e-cigarettes into Regulation of Shanghai Municipality on smoking control**							
Support	81.0	71.0–90.9	86.1	83.5–88.6	85.7	83.2–88.2	0.264
Do not support/do not know	19.0	9.1–29.0	13.9	11.4–16.5	14.3	11.8–16.8	

Among the respondents who had heard of e-cigarettes, 85.7% supported the incorporation of e-cigarettes into the regulation of the Shanghai Municipality on smoking control (Table 4). There was no statistically significant difference in the support rate between ever and never e-cigarette users (p=0.264). Controlling for gender, age, education level, occupation, income, smoking status, and perceived harmfulness, the adjusted odds ratio of support to incorporate e-cigarettes into the regulation of Shanghai Municipality on smoking control was greater among those perceiving ‘e-cigarettes and traditional cigarettes are similarly harmful’ (AOR=2.2; 95% CI: 1.4–3.5, p=0.0016) and ‘e-cigarettes are more harmful’ (AOR=3.9; 95% CI: 1.8–8.6, p=0.0008) than those who responded, ‘e-cigarettes are less harmful’.

### Use of e-cigarettes among adult residents in Shanghai

An estimated 1.3% (95% CI 0.6–1.9) of adults aged ≥15 years currently used e-cigarettes, with 2.2% of males and 0.3% of females (p=0.0045). Among the current e-cigarette users, 85.8% used both e-cigarettes and traditional cigarettes. Younger residents were more likely to use e-cigarettes, ranging from 4.1% among people aged 15–24 years to 0.3% among people aged ≥65 years (p=0.0006). Current smokers were more likely to use e-cigarettes than never smokers (p=0.0001) (Table 2).

The prevalence of ever use of e-cigarettes was 5.8% (95% CI: 4.5–7.1), and the prevalence of e-cigarette use in the past 12 months was 2.6% (95% CI: 1.7–3.6), both of which were higher among males than females (p<0.0001). Younger residents were more likely to have used e-cigarettes in the last 12 months and have ever used e-cigarettes (p=0.0001, p=0.002). Current and former smokers were more likely to have used e-cigarettes than never smokers (p<0.0001) (Table 2). After controlling for demographic characteristics and smoking status, being aged 15–24 and 25–44 years was associated with a higher rate of e-cigarette ever use. Compared to never smokers, current smokers and former smokers had, respectively, 62.9 (95% CI: 19.2–206.0) and 29.7 (95% CI: 7.9–112.0) times greater odds of having ever used e-cigarettes. After controlling for demographic characteristics, smoking status, and perceived harmfulness, respondents who perceived e-cigarettes as more harmful than traditional cigarettes were less likely to have ever used e-cigarettes compared to those who perceived e-cigarettes as less harmful (AOR=0.2; 95% CI: 0.1–0.5, p=0.0015) (Table 3).

## DISCUSSION

To the best of our knowledge, this is the first study to report e-cigarette awareness and prevalence among adults in Shanghai. Perceived harm and public support for incorporating e-cigarettes into the regulation of smoking control in the Shanghai Municipality were also explored. Our study also provides baseline data for evaluating the effect of the amended regulations of Shanghai Municipality on smoking control in public places. The key finding of this study was that those who perceived e-cigarette use as more harmful were less likely to be e-cigarette ever users and more likely to support incorporating e-cigarettes into the regulation of smoking control, compared to individuals who perceived e-cigarette use as less harmful than traditional cigarettes.

More than 70% of participants in our study were aware of e-cigarette use. The rates of current e-cigarette use, use in the last 12 months, and ever use were 1.3%, 2.6%, and 5.8%, respectively. The prevalence of e-cigarette use in our study was similar to the findings of previous studies in China but lower than the reported prevalence in some developed countries^14,16,21,22^. Our study showed a higher level of awareness of e-cigarettes. In the 2018 China Global Adult Tobacco Survey (GATS), the rate of e-cigarette awareness, the prevalence of current e-cigarette use, in the last 12 months, and ever use among urban residents was 56.3%, 1.1%, 2.5%, and 5.3%, respectively^14^. Results from five representative citywide surveys conducted from 2017 to 2018 revealed that awareness of e-cigarette products was 51.3% among urban adults in five cities, ranging from 45.0% in Chongqing to 58.7% in Xi’an. Furthermore, 4.8% of respondents reported using e-cigarettes, ranging from 3.7% in Wuhan to 6.6% in Xi’an. Approximately 0.9% reported using e-cigarettes in the past 30 days, ranging from 0.6% in Chongqing to 1.7% in Xi’an^16^. Our study found that among current users of electronic cigarettes, 85.8% were dual users. It was consistent with findings from previous studies in China^14,17^, but much higher than that in western populations^22,23^. The most likely reason for using e-cigarettes was smoking cessation^14^.

Our study found that the rates of e-cigarette awareness and use were higher among people aged <45 years than among other age groups, similar to the results of previous studies^14,16,17^. These results raise public health concerns among young adults. A report commissioned by Public Health England reveals that 61.9% of youth perceive e-cigarettes as less harmful than cigarettes^24^. A meta-analysis study showed that, in comparison to non-users, young people who were ever e-cigarette users were two times more likely to perceive e-cigarettes as less harmful than tobacco cigarettes (OR=2.01; 95% CI: 1.47–2.75)^25^. Furthermore, growing research indicates that the use of e-cigarettes may be associated with an increased risk of progression to conventional cigarette smoking in never smokers^26,27^. A previous study among university students in Shanghai showed that the most common reason for e-cigarette ever use was ‘less harmful than traditional cigarettes’ (55.0%)^28^. Our results reveal that the rate of perceiving e-cigarettes as less harmful than traditional cigarettes among young people aged 15–24 years was 55.6%. Moreover, our study found that adult residents who perceived e-cigarettes as more harmful than traditional cigarettes were less likely to have used e-cigarettes than those who perceived e-cigarettes as less harmful. A meta-analysis involving 91051 adolescents and young adults showed that e-cigarette use was associated with a higher risk of smoking intentions^27^. Another meta-analysis based on 89076 participants showed that the AOR of smoking initiation among e-cigarette users at baseline compared with non-e-cigarette users at baseline was 3.37 (95% CI: 2.68–4.24)^29^. Previous evidence indicates gateway effects from e-cigarette smoking to conventional cigarette smoking. This suggests that public health practitioners should be concerned about the perception of harm from e-cigarettes to reduce the potential hazards of e-cigarette use.

The media is the most accessible information source for the public, shaping their perceptions of health issues^30,31^. Content analysis of the full text of 639 news articles in mainland Chinese newspapers from 2004 to 2019 found that ‘e-cigarettes are less harmful than traditional cigarettes’ is the dominant argument compared with the opposite argument that ‘e-cigarettes are as harmful or more harmful than traditional cigarettes’ within the topics of relative health impact compared to cigarettes^31^. A previous mixed-methods study on adult e-cigarette users in China found that some participants received e-cigarette related information from news reported by the official media. Some participants saw television or internet news reports stating that e-cigarettes were harmful, thus raising doubts about e-cigarette safety^32^. Consistent with previous research, our study found that public media, such as the Internet and television, are the main information sources of e-cigarettes in China^14,33^. These findings suggest that health educators should provide the public with updated health knowledge about e-cigarettes through popular social media platforms.

Government policies significantly influence Chinese media coverage of relevant issues. The increased reporting of policies unfavorable to e-cigarettes aligns with the growing number of regulations restricting e-cigarettes^31^. Currently, the Shanghai Municipality Regulation on smoking control in public places, bans e-cigarette use in smoke-free venues. Our findings provide strong public support for a ban on e-cigarette use in smoke-free venues in Shanghai. However, more efforts are needed to reinforce the governance of e-cigarette use. Media reports on the harmfulness of e-cigarettes and policies regarding e-cigarettes may underpin the strong support for regulation.

### Limitations

This study has several limitations. First, our survey was a household survey, excluding those who lived collectively in places such as student dormitories, nursing homes, and military camps; there may be a few college students covered by the survey^14^. The prevalence of e-cigarette use among people aged 15–24 years, as shown in our study, may have been underestimated. Second, this was a cross-sectional survey, which precludes causal inferences. Third, this study did not assess the factors associated with current e-cigarette use, considering the sample size. Finally, given that the data used in this study were collected in 2019, temporal differences may exist.

## CONCLUSIONS

Our findings revealed that awareness of e-cigarettes was high, and the prevalence of e-cigarette use in our study was similar to findings from previous studies in China. The harmful perceptions of e-cigarettes warrant further attention from public health practitioners. Our study provides baseline data for the evaluation of the effect of the amended Regulation of the Shanghai Municipality on smoking control in public places. Future studies on e-cigarette use in Shanghai are critical for examining trends in prevalence and the potential effects of regulations.

## Data Availability

The data supporting this research are available from the authors on reasonable request.
